# Effects of lower-limb intermittent pneumatic compression on sports recovery: A systematic review and meta-analysis

**DOI:** 10.5114/biolsport.2024.133665

**Published:** 2024-06-17

**Authors:** Filipe Maia, Fábio Yuzo Nakamura, Hugo Sarmento, Rui Marcelino, João Ribeiro

**Affiliations:** 1 Research Center in Sports Sciences, Health Sciences and Human Development (CIDESD), Maia, Portugal; 2 University of Maia, Maia, Portugal; 3 Research Unit for Sport and Physical Activity (CIDAF), Faculty of Sport Sciences and Physical Education, University of Coimbra, Coimbra, Portugal; 4 Research Center in Sports Sciences, Health Sciences and Human Development (CIDESD), CreativeLab Research Community, Vila Real, Portugal; 5 Portugal Football School, Portuguese Football Federation, Oeiras, Portugal; 6 Department of Performance Optimization (GOD), Sporting Clube de Braga SAD, Braga, Portugal; 7 SC Braga Education, Braga, Portugal

**Keywords:** Athletic Performance, Physical exercise, Fatigue, Compression Therapy, Sports

## Abstract

Intermittent pneumatic compression (IPC) applied to lower limbs is becoming a popular postexercise recovery technique; however, it still lacks strong scientific support. The purpose of this systematic review and meta-analysis was to analyse the effects of lower-limb IPC on sports recovery, as well as to identify the most used protocols to optimize it. A systematic search was conducted across athletic and healthy populations, following the PRISMA guidelines, covering the databases: PubMed, Web of Science, SportDiscus, Academic Search Complete, and Science Direct; using the search terms: (“Pneumatic compression” OR “Intermittent pneumatic compression” OR “Recov* boot*”) AND (Recover*). Data was extracted, and standardized mean differences were calculated with 95% confidence and prediction interval. The pooled data analysis was conducted using a random-effects model, with heterogeneity assessed using I^2^. A total of 17 studies (319 participants) were included. The studies’ methodological quality was assessed using the PEDro scale, ranging from fair to good. Results indicate a trivial to small benefit towards lower-limb IPC in enhancing muscular function, as well as a trivial to moderate effect for pain and soreness measurements, and a highly variable effect on muscle damage markers. Moreover, protocols of about 20 to 30 minutes and pressures of about 80 mmHg appear to be the most used option to optimize recovery. In summary, lower-limb IPC might be a method with potential effects for recovery in sports, mainly reducing perceived soreness.

## INTRODUCTION

Physical capacities are fundamental to perform in sports [1]. In fact, athletes’ physical readiness influences technical and tactical behaviours, which, in turn, determine the final outcome [2, 3]. Over the last years, it is noticeable that competitive sports are becoming more and more demanding: e.g., total covered distances, as well as high-intensity actions tend to increase in competitive team sports [4]. Moreover, athletes are experiencing higher loads due to the increase in competitive density (i.e., congested fixtures) [3, 5, 6]. This fact might increase the risk of overuse and non-contact musculoskeletal injuries [7, 8]. Injuries usually force athletes to withdraw from competitions and disturb or interrupt training routines, causing a loss in physical performance [9]. Aiming to moderate constraints associated with reduced physical capacity and incidence of injuries, the need for post-match and post-training recovery strategies is increasing, imposing an optimization of the associated processes [10].

Recovery is considered a multifaceted restorative process relative to time, disturbed by internal and external factors, and dependent on reducing, altering, or breaking down from stress [11, 12]. The use of post-exercise recovery strategies is particularly required when there is a need to limit the severity of fatigue and shorten its duration [13, 14]. For instance, in sports such as swimming, track and field, or powerlifting, athletes typically participate in more than one event in the same session or day, separated by minutes or hours, while multiday races characterize sports like road cycling or ultramarathon [15–17]. On the other hand, in sports such as soccer, basketball or baseball, athletes typically compete in congested calendars [18]. Recovery modalities are highly trendy and not always fully supported by scientific literature with regard to their purported effects [19]. Intermittent Pneumatic Compression (IPC) is an emerging method, grown in popularity among practitioners and athletes over the past few years [20]. This method consists of pumped inflation and deflation pressure cycles of air bladders, usually applied on the lower limbs. Lower-limb IPC is performed using sleeves that cover the feet, calves, or whole thighs and legs. These cycles can be performed uniformly or in sequence, at different pressures and cycle rates [21].

Despite being novel in sports, this technology has been used in medical care units since 1970 [22]. In this area, IPC is widely utilized to prevent stasis and deep vein thrombosis, and to treat lymphedema and leg ulcers [23–28]. Although these devices are shared between sport and medical areas, the applied protocols tend to vary, mainly in pressure and duration of application [29]. In sports science, this method remains controversial, with the majority of studies reporting either advantages [20, 30, 31] or a neutral effect [32, 33] for different recovery parameters of muscular function, perceptual measures and physiological markers. The effect of compression provided by these devices would, in theory, increase local blood flow, which is associated with an acceleration of the removal rate (outflow) of metabolic by-products that could adversely affect physical performance [34]. Additionally, the induced compression may also assist in reducing oedema, through the limitation of the space available for swelling to form, therefore restricting the inflammatory and muscle damage responses [35]. Intermittent pneumatic compression devices are lightweight and thus highly portable and do not require additional human resources (i.e., it can be performed by the athletes themselves), simplifying and making the intervention more accessible [36]. Therefore, IPC may be a promising practical solution for recovery in sports, if it proves to be effective.

Although the organization and systematization of the evidence concerning the use of this recovery method is incipient, athletes seem to have embraced IPC by regularly using it [37, 38]. Therefore, it becomes important to elucidate its effects and search for the most effective recovery conditions. The purpose of this study is to address this important gap in the scientific evidence by systematically and meta-analytically reviewing the effects of lower-limb IPC, in the context of sports recovery, on muscular function, subjective measures, and physiological markers, as well as explore the most used recovery protocols.

## MATERIALS AND METHODS

### Search Strategy: Databases and inclusion criteria

**FIG. 1:**
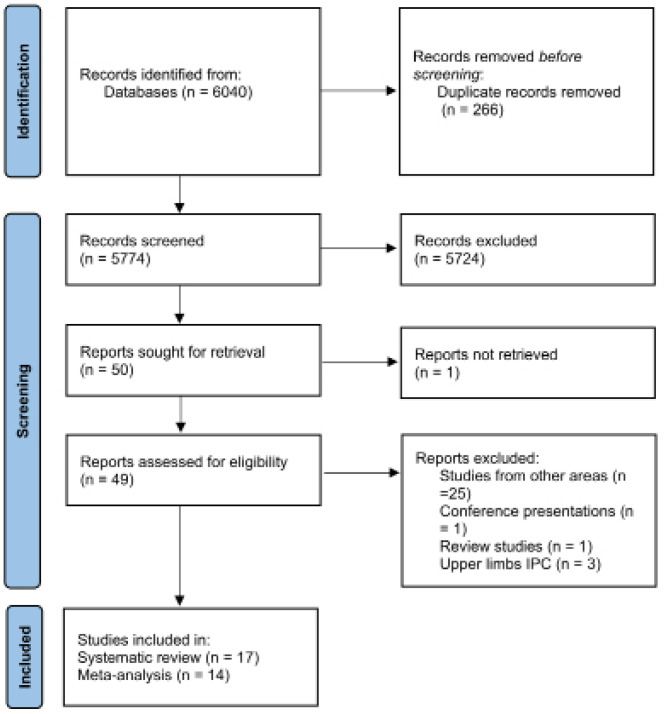
PRISMA Flow Diagram of the procedures used for the articles search.

This systematic literature review with meta-analysis was conducted following the Preferred Reporting Items for Systematic Reviews and Meta-Analysis (PRISMA) guidelines [39]. The study protocol was registered at the International Prospective Register of Systematic Reviews (PROSPERO) under the code CRD42021273598 (Figure 1).

The extensive literature search covered the following electronic databases: PubMed, Web of Science (all databases), SportDiscus, Academic Search Complete, and Science Direct. All articles published until 1^st^ November 2022 were considered. The searched keywords were: (“Pneumatic compression” OR “Intermittent pneumatic compression” OR “Recov* boot*”) AND (Recover*).

The criteria of selection were established according to the PICOS strategy, as follows:

– *Participants:* Adults (age ≥ 18); male and female; healthy and physically active or athletes.
– *Intervention:* Articles that reported a fatiguing activity, a recovery protocol using IPC, and subsequent measurements of recovery.
– *Comparator:* Studies comparing the use of IPC with placebos or passive rest conditions.
– *Outcomes:* Recovery parameters: neuromuscular function (e.g., time trial performance, maximal voluntary contraction), subjective measures (e.g., perceived fatigue, perceived soreness), or physiological markers (e.g., creatine kinase, blood lactate) of recovery, measured up to 96 hours after the fatiguing protocol.
– *Study Design:* Randomized controlled trials (with parallel groups or crossover designs).

Articles were excluded if they: (i) reported IPC treatments after surgery, recovery from diseases, or injuries treatment; (ii) reported results of unhealthy populations; (iii) animal studies; (iv) reported results unrelated to practical post-exercise recovery strategies; (v) reported upper limbs IPC interventions; (vi) poster presentations, articles composed only by abstracts or review studies; and (vii) studies written in a language other than English. After excluding articles by title, the second screening for the inclusion of studies was conducted based on abstract analysis and full text versions. This process was conducted by two investigators independently (FM, JR), and discrepancies were solved by a third investigator (FYN).

### Quality of Studies and Risk of Bias

Two investigators (FM, HS) assessed the overall studies’ methodological quality independently, following the criteria of the Physiotherapy Evidence Database (PEDro) scale. The scale consists of 11 items encompassing external validity (item 1), internal validity (items 2 to 9), and statistical reporting (items 10 to 11) [40].

Publication bias was assessed by visually analysing funnel plots and formal testing for funnel plot asymmetries using the “trim and fill” algorithm [41, 42].

### Data extraction and analysis

All meta-analysis calculations and graphical designs were conducted using the software IBM SPSS Statistics for Windows, version 28 (IBM Corp., Armonk, N.Y., USA). General characteristics and results of the individual studies were extracted independently by two authors (FM, JR). In case of a lack of information, the statistics were extrapolated from the published figures and graphs, through the GetData Graph Digitizer software for Windows, version 2.26.0.20 [43]. A meta-analysis with a random-effects model was performed using the restricted maximum-likelihood estimation, to examine the effects of lower-limb IPC on functional, pain and soreness and muscle damage measurements. Summary effect sizes (ES) were presented for each study using standardized mean difference (SMD) to compare the effects between lower-limb IPC and control conditions. The ES are expressed as Hedges’ g, to account for possible overestimation of the true population ES in small studies. The magnitude of ES was interpreted according to the following scale: < 0.20 = negligible effect, 0.20–0.49 = small effect, 0.50–0.79 = moderate effect, > 0.80 = large effect [44]. A *p*-value of 0.05 was considered statistically significant for all analyses and a 95% confidence interval (CI) and prediction interval (PI) were assumed. To combine results from parallel-group and crossover trials, appropriate formulae was used for standardized mean ES and standard errors [45]. For the purposes of meta-analysis, the direction of change for some measures was reversed to ensure consistency of directionality across the tests (e.g., reduced time trial times indicate improvement in recovery, whereas higher jump height also indicates improved recovery). When dealing with multiple outcomes for each subgroup, the recommendations of Moeyaert et al. [46] were followed. Heterogeneity was assessed using the *I*^2^ statistic, which describes the percentage of variability in effect estimates attributable to heterogeneity rather than chance [47]. *I*^2^ values of 25%, 50% and 75% can be considered to reflect small, moderate and large degrees of heterogeneity [48]. To provide some criterion of temporal echo-coherence consistency, subgroup analysis was carried out, grouping studies into the following time points: 0–2, 24, 48, 72 and 96 hours post IPC protocol.

## RESULTS

### Search, Selection, and Inclusion of Publications

The initial search identified 6040 articles that were exported to the citation manager software EndNote™20 (Clarivate Analytics, Philadelphia, PA). Duplicate references were removed (n=266), resulting in 5774 articles. Those remaining manuscripts were screened by title (excluding 5724). From the 50 articles left, the analyses of the abstract and full text (except one not retrieved) were carried out. A total of 17 articles were included in the systematic review, and from those 14 were included in meta-analysis. All excluded records did not match the inclusion criteria previously described or were not retrieved.

### Quality assessment

The quality of the 17 studies included in this review ranged from a score of 5 to 8, according to PEDro scale, with a mean of 6.29 and a standard deviation of 0.67, indicating an overall good methodological quality [40]. As illustrated in Table 1, all studies adequately reported a baseline comparison, provided adequate follow-ups (considering dropouts ratio), intention to treat analysis, between groups statistical comparisons, and reported point and variability measures. Additionally, most of the studies reported a random allocation (88%), and few reported the blinding of participants to procedures (35%). Finally, none of the studies reported allocation concealment, as well as blinding to therapists and assessors.

**TABLE 1:** Study quality according to Physiotherapy Evidence Database (PEDro) scale.

Study	Eligibility criteria and source	Random allocation	Concealed allocation	Baseline comparability	Blinding of participants	Blinding of therapists	Blinding of assessors	Adequate follow-up	Intention-to-treat analysis	Between-group statistical comparisons	Reporting of point and variability measures	Total^*^
Wiecha et al. [49]	1	1	1	1	1	0	0	1	1	1	1	8 (Good)
Khan, Ahmad and Hussain [50]	1	1	0	1	1	0	0	1	1	1	1	7 (Good)
Draper et al. [33]	1	1	0	1	0	0	0	1	1	1	1	6 (Good)
Collins et al. [51]	1	1	0	1	0	0	0	1	1	1	1	6 (Good)
Marcello, Fortini and Greer [52]	1	0	0	1	1	0	0	1	1	1	1	6 (Good)
Heapy et al. [30]	1	1	0	1	0	0	0	1	1	1	1	6 (Good)
Northey et al. [53]	1	1	0	1	0	0	0	1	1	1	1	6 (Good)
Overmayer and Driller [54]	1	1	0	1	0	0	0	1	1	1	1	6 (Good)
Haun et al. [55]	1	1	0	1	1	0	0	1	1	1	1	7 (Good)
Haun et al. [56]	1	1	0	1	1	0	0	1	1	1	1	7 (Good)
Hoffman et al. [20]	1	1	0	1	0	0	0	1	1	1	1	6 (Good)
Keck et al. [57]	1	1	0	1	0	0	0	1	1	1	1	6 (Good)
Martin et al. [58]	1	1	0	1	1	0	0	1	1	1	1	7 (Good)
O’Donnell and Driller [32]	1	1	0	1	0	0	0	1	1	1	1	6 (Good)
Sands et al. [31]	1	1	0	1	0	0	0	1	1	1	1	6 (Good)
Cochrane et al. [59]	1	1	0	1	0	0	0	1	1	1	1	6 (Good)
Waller, Caine and Morris [21]	1	0	0	1	0	0	0	1	1	1	1	5 (Fair)

* Total score from a possible maximum of ten. Scores of: < 4 are considered ‘poor’; 4 to 5 are considered ‘fair’; 6 to 8 are considered ‘good’ and 9 to 10 are considered ‘excellent’ [40].

**FIG. 2:**
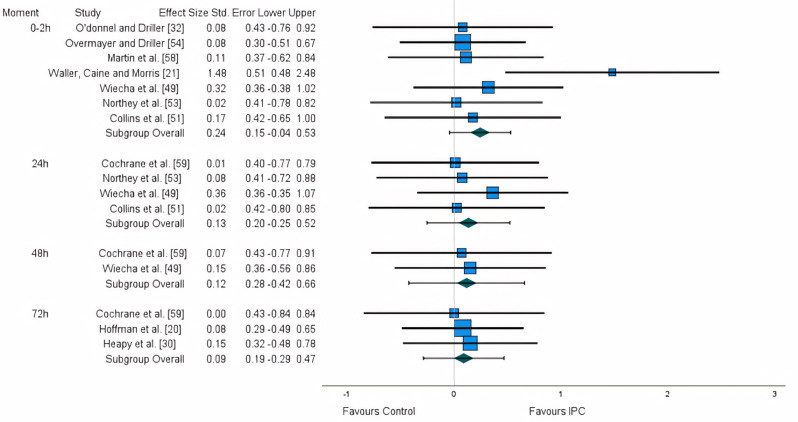
Forest plot illustrating the effect of IPC on functional measures.

### General description of the studies

From the 17 studies included in this review, 12 assessed muscular function and performance parameters [20, 21, 30, 32, 49, 51, 53–56, 58, 59], 12 assessed subjective measures [20, 21, 30–33, 49, 51, 53–56], and 12 assessed physiological markers [32, 33, 49–52, 54–59] (Table 2). The synthesis of the meta-analysis results is displayed in Table 3.

**TABLE 2:** Summary of the randomized controlled trials included in the review.

Study	Age, Sport/Condition and sex	Design	N	Experimental design	Main results
Wiecha et al. [49]	Age: 22.05 ± 3.3 yrs Population: Healthy active Sex: Male	Parallel group	30	Exercise 5 × 20 box drops with vertical jump. Recovery protocol: 30 min peristaltic IPC at 80 mmHg. Recovery parameters: Physiological (muscle damage via serum CK), subjective (perceived pain), and functional measures (range of motion and muscle strength – 5 series knee extension and flexion (0°–90°), at 60° · s^−1^).	IPC had no influence on DOMS, ROM, strength, and muscle damage markers (CK).

Khan, Ahmad and Hussain [50]	Age: 18 to 25 yrs Population: Collegiate basketball players Sex: Male	Crossover	16	Exercise: Submaximal treadmill running Recovery protocol: 15 min peristaltic IPC at 80 mmHg Recovery parameters: Physiological (HR)	IPC is effective in lowering the heart rate during the first minute of recovery after submaximal exercise.

Draper et al. [33]	Age: 38.7 ± 11.2 yrs Population: Distance runners Sex: Male and female	Crossover	10	Exercise: 20-mile run at 70% or more of $\dot{\text{V}}$O_2max_. Recovery Protocol: 1 h sequential IPC from 90 mmHg to 100 mmHg of pressure. Recovery Parameters: Physiological (inflammation via CRP) and subjective measures (perceived pain).	IPC offered little to no benefit in recovery from a prolonged bout of running. No effect of treatment on CRP.

Collins et al. [51]	Age: 21.6 ± 3.4 Population: Team sport athletes Sex: Male	Parallel group	21	Exercise: Cycles of maximal CMJ, a ‘20-metre sprint out’ and a ‘20-metre sprint back’ Recovery protocol: 20 minutes sequential at 235 mmHg Recovery Parameters: Functional (CMJ, dynamometry – 4 maximal extensions at 60, 120 and 180 °/s, with 2 min of rest separating each velocity + 2 maximal extensions and 2 flexions were completed isometrically), subjective (perceived muscle soreness), physiological (CK).	Following high-intensity exercise, IPC has potentially beneficial effects upon biomarkers of recovery, without affecting the neuromuscular function.

Marcello, Fortini and Greer [52]	Age: 16 to 60 yrs Population: Cyclists Sex: Male and female	Crossover	10	Exercise: 2 × 60 minute steady state rides and 30 minutes recovery period between rides. Recovery Protocol: ~25 minutes peristaltic IPC at 80 mmHg. Recovery Parameters: Physiological (HR and BLa).	No influence on HR and cadence. IPC promoted either an increase in lactate production or an impairment in clearance.

Heapy et al. [30]	Age: 41 ± 8 yrs Population: Ultra-marathon runners Sex: Male and female	Parallel group	37	Exercise: Ultra-marathon competitive event. Recovery Protocol: 20 min lower extremity treatment and peristaltic compression up to 80 mmHg. Recovery Parameters: Subjective (lower body muscle pain, soreness rating and overall muscular fatigue score) and functional measurements (400 m runs).	IPC resulted in improvements in overall muscular fatigue. IPC provided some immediate subjective improvements in muscle fatigue, pain and soreness. No effect neither in 400 m run time nor both functional and subjective measures of fatigue and soreness after 96 hours.

Overmayer and Driller [54]	Male age: 40 ± 14 yrs Female age: 29 ± 12 yrs Population: Trained cyclists	Crossover	21	Exercise: 20 min TT on cycle ergometer. Recovery Protocol: 30 min peristaltic IPC treatment at 80 mmHg. Recovery Parameters: Subjective (perceived recovery, RPE), physiological (Max HR, BLa), and function (4 min TT) measures.	Max HR was higher in IPC at the 4-min TT. IPC had no effect on performance or recovery, mean HR, RPE and average power. Unclear effect for pre to post recovery BLa concentration. IPC enhanced TQR.

Haun et al. [55]	Age: 21.6 ± 2.4 yrs Population: Healthy endurance trained Sex: Male	Parallel group	18	Exercise: HIIT (22.5 min in duration - 15 rounds running for 45 s and walking for 45 s). Recovery Protocol: 1 h peristaltic EPC treatment at ~70 mmHg. Recovery Parameters: Functional (6 km run time trial and flexibility), physiological (muscle damage via serum CK; inflammation via CRP and oxidative stress via 8-isoprostane) and subjective measures (muscle soreness via PPT).	No significant differences in 6 km TT, flexibility, muscle damage, oxidative stress and inflammation markers, and muscular soreness. EPC treatment of the lower limbs reduced markers of oxidative stress in skeletal muscle tissue.

Haun et al. [56]	Age: 22.5 ± 0.8 yrs Population: Healthy resistance-trained males Sex: Male	Parallel group	20	Exercise: 3 consecutive days of heavy and high-volume RT. Recovery Protocol: 1 h peristaltic EPC at 70 mmHg. Recovery Parameters: Functional (1RM, flexibility), subjective (PPT), and physiological measures (muscle damage via CK, inflammation via IL-6 and CRP, and RNA expression).	EPC improved flexibility and reduced PPT on days 3–7. No effect on lifting volume and CK. Larger reduction on CRP and IL-6. EPC reduced muscle proteolysis, oxidative stress, and muscle soreness.

Hoffman et al. [20]	Age: 43 ± 8 yrs Population: Ultra-marathon runners Sex: Male and female	Parallel group	47	Exercise: Ultra-marathon competitive event. Recovery Protocol: 20 min interventions with peristaltic compression of 80 mmHg. Recovery Parameters: Subjective (lower body muscle pain, soreness rating and overall muscular fatigue) and functional measures (400 m runs).	IPC provided some immediate subjective benefits. IPC improved overall muscular fatigue score. No effect on functional measures.

Northey et al. [53]	Age: 24 ± 6.3 yrs Population: Strength trained participants Sex: Male	Crossover	12	Exercise: 10 sets with 10 repetitions of back squats at 70% 1 repetition maximum with 3-minute rest between sets. Recovery Protocol: 45-minute sequential IPC at 80 mmHg. Recovery parameters: Functional (peak torque – 5 maximal voluntary contractions at 30º · s^−1^, with 30 s of rest, CMJ and SJ), and subjective (perceived soreness and recovery).	There were no significant differences between conditions for any of the postexercise measures.

Keck et al. [57]	Age: 28.1 ± 7.3 yrs Population: Cyclists Sex: Male	Crossover	10	Exercise: 90-minute cycling session (warm up + 10 intervals (2 minutes at 80% watt max followed by 4 minutes at 50% watt max) + cool down). Recovery Protocol: 2 × 60 min (1 h apart) of peristaltic IPC at 70 mmHg. Recovery Parameters: Physiological biomarkers (muscle glycogen, blood glucose, insulin, and BLa) and HR measures.	No effect on muscle glycogen, plasma glucose, plasma insulin, BLa and heart rate.

Martin et al. [58]	Age: 22.73 ± 4.05 yrs Population: Healthy active participants Sex: Male and female	Crossover	14	Exercise: 2 Wingate Anaerobic test (3 minutes rest) + Recovery + 1 Wingate Anaerobic test. Recovery Protocol: 30 minutes peristaltic EPC at ~70 mmHg. Recovery Parameters: Functional (peak power, mean power, and fatigue index – Wingate Anaerobic test), and physiological (HR and BLa).	EPC improved BLa clearance. No effect on HR and performance parameters. No significant effect on anaerobic performance.

O’Donnell and Driller [32]	Age: 29 ± 9 yrs Population: Triathletes Sex: Male	Crossover	10	Exercise: Participants performed a cycling HIIT session – Recovery – 5 km TT run. Recovery Protocol: 30 minutes peristaltic IPC device at 80 mmHg. Recovery Parameters: Subjective (TQR and RPE), physiological (HR and BLa) and functional measures (5 km run TT).	No effect/trivial on performance. No differences in blood lactate. Unclear results on TQR.

Sands et al. [31]	Age: 18–40 yrs Population: Internationally competitive athletes Sex: Male and female	Parallel group	24	Exercise: Morning and afternoon practice. Recovery Protocol: 15 minutes peristaltic IPC treatment at a pressure ranging from 70 mmHg to 80 mmHg. Treatment performed between morning and afternoon practice. Recovery Parameters: Subjective measures (PPT).	IPC seemed to enhance recovery by the reduction of muscle tenderness. IPC resulted in increased values of PPT.

Cochrane et al. [59]	Age: 21.0 ± 1.7 yrs Population: Healthy and physically active individuals Sex: Male	Crossover	10	Exercise: Muscle Dynamometry – 3 × 100 ECC contractions. Recovery Protocol: 30 minutes peristaltic IPC from 60 to 80 mmHg. Recovery Parameters: Physiological (muscle damage via CK), and functional (single leg VJ and muscle dynamometry – eccentric, concentric, and isometric: 3 RM were separated by 10 s rest and a 2-min rest between contraction type).	No differences were observed in dynamometry and VJ, muscle recovery, and force loss in isokinetic contractions.

Waller, Caine and Morris [21]	Age: 25.2 ± 1.72 yrs Population: Healthy participants Sex: Male	Crossover	9	Exercise: Set of shuttle runs for one hour. Recovery Protocol: 1 h sequential IPC (70:65:60 mmHg). Recovery Parameters: Functional (VJ) and subjective measures (soreness diagram).	IPC treatment produced a smaller reduction on VJ. Perceived soreness was reduced by IPC until 48 hours. No effect on calf and thigh circumferences.

Notes: BLa – Blood lactate; CK – Creatine Kinase; CMJ – Countermovement jump; CRP – C Reactive Protein; DOMS – Delayed Onset of Muscle Soreness; ECC – eccentric; EPC – External Pneumatic Compression; HIIT – High Intensity Interval Training; HR – Heart Rate; IL-6 – Interleukin 6; IPC – Intermittent Pneumatic Compression; mmHg – millimetre(s) of mercury; PPT – Pressure Pain Threshold; RM – Maximum Repetition; RPE – Rated Perceived Exertion; RT – Resistance training; SJ – Squat jump; TQR – Total Quality Recovery; TT – Time Trial; VJ – Vertical Jump; $\dot{\text{V}}$O_2max_ – Maximal Oxygen Uptake.

**TABLE 3:** Meta-analysis summary.

Outcome	SMD (95% CI)	95% PI	p-value	Z	Std. Error	I^2^
*Muscle function 0–2 h*	0.243 (-0.43; 0.528)	-0.134; 0.620	0.096	1.665	0.146	0.2%
*Muscle function 24 h*	0.134 (-0.253; 0.521)	-0.716; 0.984	0.498	0.678	0.198	0%
*Muscle function 48 h*	0.117 (-0.424; 0.658)	-^*^	0.672	0.424	0.276	0%
*Muscle function 72 h*	0.089 (-0.287; 0.466)	-2.353; 2.532	0.642	0.464	0.192	0%
*Pain and Soreness 0–2 h*	0.486 (0.117; 0.855)	-0.367; 1.339	0.010	2.580	0.188	20.4%
*Pain and Soreness 24 h*	0.344 (-0.048;0.736)	-0.711; 1.399	0.085	1.720	0.200	47.0%
*Pain and Soreness 48 h*	0.644 (-0.012; 1.300)	-1.536; 2.824	0.055	1.923	0.335	79.6%
*Pain and Soreness 72 h*	0.169 (-0.151; 0.490)	-0.351; 0.689	0.301	1.035	0.164	0%
*Pain and Soreness 96 h*	0.368 (0.045; 0.691)	-0.157; 0.893	0.026	2.231	0.165	0%
*CK 24 h*	-0.203 (-0.655; 0.249)	-3.134; 2.728	0.378	-0.882	0.231	0%
*CK 48 h*	0.873 (-0.427; 2.173)	-5.169; 6.914	0.188	1.316	0.663	88.3%
*CK 72 h*	0.100 (-0.996; 1.197)	-12.894; 13.095	0.329	0.179	0.559	78.1%

SMD – Standardized Mean Difference; 95% CI – 95% Confidence Interval; 95% PI – 95% Prediction Interval; Std. Error – Standard Error;

* to calculate the prediction interval, a minimum of 3 studies are required.

### Effects on functional and performance measures

Functional measures are particularly important, as they are directly related to the athletes’ performance. For this purpose, and considering the reduced number of studies, a variety of measures of similar nature were grouped: time trial [20, 30, 54], maximum voluntary contraction (MVC) [49, 51, 53, 56, 59], and power output [21, 51, 53, 58, 59]. A non-significant small effect was found for the first 2 hours (ES:0.243). A trivial effect was found for 24 h (ES: 0.134), 48 h (ES: 0.117), and 72 h (ES: 0.089) (Figure 2). After the application of the trim and fill method, the adjusted values remained unaltered.

Flexibility and range of motion (ROM) were not considered for meta-analytical purposes, as these variables highly differ from the remaining. From the three studies [49, 55, 56] assessing the effects of lower-limb IPC on these measures, two studies reported a lack of significant differences [49, 55], while one detected a moderate effect favouring the use of lower-limb IPC [56].

### Effects on subjective measures

From the 12 studies assessing subjective measures, 10 reported outcomes of pain and soreness [20, 21, 30, 31, 33, 49, 51, 53, 55, 56]. The vast majority of studies measured pain and soreness daily, immediately after the recovery protocol and at least for 96 h [20, 30, 33, 60]. However, two studies started the measurements of pain and soreness at the 48 h time point [55, 56], two measured these variables within 48 h [21, 49], two within 24 h [51, 53], and one only presented results immediately after the recovery protocol [31].

**FIG. 3:**
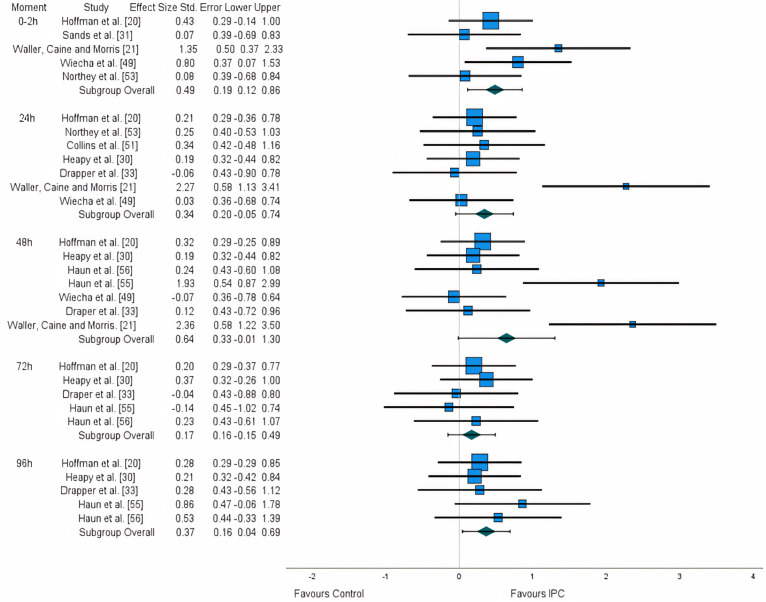
Forest plot of the meta-analysis illustrating the effect of IPC on pain and soreness.

**FIG. 4:**
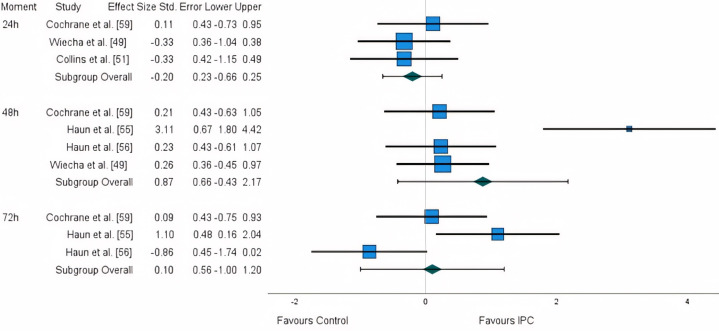
Forest plot illustrating the effect of IPC on creatine-kinase concentrations.

A significant small effect was observed for the 0–2 h time interval (ES: 0.486), as well as for the 96 h time frame (ES:0.368). A non-significant small effect was also observed for the 24 h time point (ES: 0.344), a moderate effect for the 48-hour (ES: 0.644), and a trivial effect for the 72 h (ES:0.169). After the application of the trim and fill method, the adjusted values remained unaltered (Figure 3).

Two studies assessed perceived fatigue [20, 30], which was measured using a visual analogue scale, and three assessed perceived recovery [32, 53, 54], using the Total Quality Recovery scale. In this regard, the use of lower-limb IPC seems to provide trivial to small benefits enhancing the perception of recovery, as well as trivial effects reducing the perception of fatigue.

### Effects on physiological markers

A great number of different physiological measures were analysed across the studies. For this reason, to ensure consistency of measures, we only considered muscle damage markers (CK) for meta-analytical purposes, along with inflammation (CRP, IL-6), anaerobic metabolism marker (BLa) and heart rate (HR) as part of the review.

Five studies measured CK: three at the 24 h time point [49, 51, 59], four at the 48 h [49, 55, 56, 59], and three at the 72 h [55, 56, 59]. A large disparity in results was identified, with studies reporting both advantages and disadvantages. A non-significant negative small effect (ES: -0.203) was observed for the first 24 h, while a large positive effect (ES: 0.873) was observed for the 48 h time point. Lastly, for the 72 h time point, a trivial non-significant effect (ES: 0.10) was identified (Figure 4). After the application of the trim and fill method, the adjusted values remained unaltered.

Inflammation markers were addressed by three studies, using two different biomarkers: CRP [33, 55, 56] and IL-6 [55, 56]. The measurements took place immediately after the recovery protocol [33, 56], and at 24 h until at least 96 h, on a daily basis [33, 55, 56]. The results also showed to be conflicting, with studies reporting advantages and disadvantages considering the use of lower-limb IPC. With the available results, no further extrapolations can be obtained.

BLa was typically measured during the recovery period, testing the ability of lower-limb IPC to improve its clearance, with two studies reporting advantages [32, 58] and two a neutral effect [54, 57], even after using similar recovery protocols. Additionally, one study [52] measured BLa on subsequent efforts, where IPC either promoted an increased production or an impairment in clearance.

Two studies [52, 54] addressed measures of mean and maximum heart rate on exercise performed after the recovery protocol, also demonstrating no significant differences. Finally, one study [50] assessed the capability of IPC to restore baseline HR values, in which a positive effect was only detected for the first minute of IPC protocol.

### Recovery protocols

Regarding the recovery protocols, a great heterogeneity was observed when considering duration, pressure, and type of application. The protocols used ranged from 15 [31] to 60 minutes [21, 33, 55–57], and the applied pressure ranged from 60 mmHg [21, 59] to approximately 235 mmHg [51]. Finally, considering the type of application, four studies [21, 33, 51, 53] applied a sequential program (i.e., air bladders inflate and deflate one at a time), while the remaining chose a peristaltic program (i.e., air bladders inflate one at a time, keeping the pressure for the whole cycle, and deflate all together).

## DISCUSSION

Competitive sports involve high levels of physiological stress, especially during in-season periods [61]. Additionally, the margins between winning and losing have been narrowing over the last decades [62]. Therefore, marginal performance gains may translate into significantly improved competitive outcomes [62]. In this sense, to improve the chances of succeeding, the recovery process plays an important role in athletic performance [59, 61]. Practical post-exercise recovery techniques are expected to produce small, but meaningful additional benefits to athletes’ recovery states, and their use should be seen as a complement to the “natural” (e.g., sleep hygiene, ordinary food) recovery process [63, 64]. The current study used a systematic review and meta-analysis approach to provide insights into the use of lower-limb IPC as a sports recovery tool.

### Functional measurements

One of the primary sought recovery outcomes while implementing recovery methods is the attenuation of post-exercise losses in athletes’ physical capacities [11, 65]. A variety of physical measures were considered in the current study, including aerobic and anaerobic power performance, muscular strength and jump height. From those, recovery with IPC devices seemed to provide non-significant small benefits on parameters associated with muscular function (e.g., peak power) on short-term (i.e., first two hours). These could be explained by the positive effect of augmenting blood flow (the theoretical benefit of IPC) on subsequent performance, as previously reported in scientific literature [35, 66]. Moreover, IPC provides mechanical pressure to the limbs, which is hypothesized to help reduce muscular stiffness and improve muscle compliance, therefore enhancing functional capacity [67]. There are other strategies with similar acting mechanisms, such as the use of compression garments or massage treatments [68–70]. Two studies compared the recovery kinetics of ultramarathon participants using IPC, a control condition, and massage treatments [20, 30], finding similar effects between IPC and massage. Additionally, one study [60] compared the use of IPC to compression garments, finding greater recovery benefits for the IPC group. More studies directly comparing IPC with similar recovery techniques could help clarify the real effectiveness of this recovery strategy. Associated with the lack of significant effect, due to the risk of bias regarding blinding procedures, and the reduced number of studies and participants, it is not certain that these results represent the “true” effects of lower-limb IPC, at this point. Future research should consider this critical aspect to reduce biases and limitations that can influence the outcomes, and test the effectiveness of IPC on functional parameters using more robust study methods.

### Subjective measures

It is recognized that subjective and objective measures of athletes’ recovery do not necessarily follow the same trend and time courses [71]. The subjective parameters seemed to be the most benefited from the use of IPC devices. Nevertheless, it should be noted that the use of a placebo condition is fundamental to limit the interference of psychological factors associated with participants’ beliefs and expectations [72]. From the 17 studies included in this review, only six were conducted using a placebo condition [49, 51, 52, 55, 56, 58]; therefore, these results should be cautiously considered.

Regarding pain and soreness measurements, the effect sizes were shown to be higher at 48 h post-exercise. This coincides with the peak values of DOMS [73] and may indicate the higher efficacy of lowerlimb IPC on the reduction of pain and soreness in this specific time frame. The mechanism behind the advantages provided by lower-limb IPC on perceptions of pain and soreness could be associated with a reduction in neuromuscular excitability, achieved by stimulating sensory receptors and decreasing the level of muscular tension [67].

It is important to highlight that athletes appear to perceive similar effectiveness between compression garments and IPC; however, they adhere more to the former, possibly due to the easier access to compression garments considering their portability and cost [37, 38]. The reviews about compression garments reveal that this method is also able to reduce post-exercise muscle soreness within 24 and 96 hours [74, 75].

Perceived recovery was assessed in three studies, revealing either small positive [32, 53] or trivial effects [54]. Accordingly, the results of the two included studies that assessed perceived fatigue reported trivial advantages towards the use of IPC [20, 30]. There is some evidence suggesting that lower-limb IPC may not expressively affect the perceptions of physical fatigue and may provide some small additional benefits concerning perceived recovery; however, we expect that future studies further investigate whether recovery perceptions are improved by this intervention while controlling for the placebo effect.

### Physiological markers

Creatine kinase is a marker of muscle damage, highly variable interand intra-individually, usually peaking approximately 48 h after highintensity exercise [56, 76, 77]. This feature leads to some limitations in our results considering that they are based only in five studies (addressing this measure), across different observation points. It should be noted that results could have been influenced by participants’ training status: physically active participants typically show a lower level of muscle damage markers compared to untrained subjects [78]. From the studies included in the CK analysis, three analysed trained participants [51, 55, 56], and two reported results of recreationally active healthy subjects [49, 59]. Moreover, participants were subjected to different exercise interventions; therefore, it is likely that different levels of muscle damage were induced, which inevitably would have generated different kinetics of serum-CK. For instance, some fatigue-inducing interventions may not have been sufficiently intense to produce expressive levels of muscle damage, which partially helps to explain the inconsistent results. Overall, since results are generally variable, with wide confidence intervals encompassing both negative and positive effects, it is unlikely that lower-limb IPC affects this marker. By the effects of lower-limb IPC on CK, it appears that post-exercise inflammation markers (CRP, IL-6) are also not affected by the use of this strategy [33, 55, 56]. More studies should take these biomarkers into consideration, to fully explore the effects of lower-limb IPC, especially after more damaging events (e.g., downhill running).

In addition, the use of lower-limb IPC seems not to affect BLa clearance. Nevertheless, it has been suggested that BLa clearance does not necessarily translate into better performances during subsequent exercise [13]. Interestingly, one study [52] reported that the use of IPC caused higher concentrations of BLa during subsequent performance, which may suggest an impairment in the clearance capacity or an improvement in the production capacity. Finally, it was expected that HR measures would kept unaltered after IPC since the former is not necessarily associated with changes in blood flow [35]. However, it is important to note that there is no clear association between HR reduction and recovery of performance indices. It is suggested that globally IPC does not affect HR responses. Regarding BLa, it is important to show in future studies whether higher values during the subsequent exercise are beneficial or not to anaerobic performance.

### Recovery protocols

The protocols applied in the included studies varied substantially regarding duration and pressure. Preliminary evidence suggests that a single bout of 20 to 30 minutes may be sufficient to provide immediate and mid-term benefits [20, 30]. The pressure used also varies across studies: it is suggested that a low-pressure treatment offers fewer benefits than high-pressure, but greater effects when compared to passive rest [21]. The results indicate that lower-limb IPC pressures of approximately 80 mmHg, sustained for 20 to 30 minutes seem to be the most used option to recover muscle mechanical functions and reduce perceptions of pain and soreness. To obtain strong and consistent evidence regarding the most contributory recovery options, future studies should consider this critical aspect by comparing and controlling the recovery kinetics following different IPC protocols.

### Quality of Evidence and Limitations

The majority of studies revealed a good methodological quality; however, the risk of bias is evident when it is not possible to blind the participants and conceal the treatment allocation [79]. In addition, a limitation of crossover trials is the carryover effect [45]. The power of the selected studies was mainly small, considering sample sizes. Moreover, the best results were found for subjective measures. Although general well-being is recognized as important for athletic performance [80], these measures are more susceptible to biases. This susceptibility arises from various factors, including the participant’s belief in the recovery technique [54], familiarity with the assessment scales [81], among others. In this regard, some of the scales assessed still lack proper scientific validation [82]. The choice of different exercise interventions, followed by different recovery protocols considering time, pressure, type of application and moment turn different results expected to occur. Moreover, given the scarcity of research on this recovery technique, it was not possible to cluster the results according to exercise interventions. To address this limitation, more studies on the topic are required. Finally, sex, competitive level or sports differences were not considered, which could limit the validity of our conclusions.

Despite the limitations acknowledged, this study provides reliable results about lower-limb IPC in the context of sports recovery, providing information that may be useful to assist coaches, athletes, and support staff when considering lower-limb IPC as a recovery tool.

## CONCLUSIONS

Performance outcomes of elite sport are discussed in the tiny detail, therefore, marginal additions to athletes’ readiness to compete may be of capital importance, considering the chances to succeed [62, 83]. The results of the present study indicate that lower-limb IPC provides (at best) moderate beneficial effects concerning perceptions of pain and soreness, as well as small, however non-significative effects on the recovery of muscle mechanical functions. Variable and unclear results were found on the clearance of muscle damage and inflammation markers, while HR and BLa removal rates seem to be unaltered with the use of lower-limb IPC. Finally, the most used recovery protocols appear to last from 20 to 30 minutes with an applied pressure of about 80 mmHg.

### Future research recommendations

Intermittent Pneumatic Compression is still in an embryonic phase of scientific evidence, which is demonstrated by the different protocols applied, and the reduced number of original studies analysing this recovery technique as a practical post-exercise strategy. The exercise intervention may be fundamental to determine the recovery response since the type of exercise (e.g., intermittent and continuous efforts) promotes different fatigue effects [84–86]. In addition, valid subjective scales, functional tests, and physiological analyses should be addressed to fully explore the potentialities of IPC. Finally, larger sample sizes, consistent follow-up timings, the use of placebo conditions, and the blinding of procedures to therapists and assessors may help prevent biases in studies assessing the effects of practical postexercise recovery strategies.

## Protocol and Registration

The study protocol was registered at the International Prospective Register of Systematic Reviews (PROSPERO) under the code CRD42021273598.
